# Form factor of prismatic particles for small-angle scattering analysis

**DOI:** 10.1107/S1600576725000676

**Published:** 2025-03-07

**Authors:** Jules Marcone, Jaime Gabriel Trazo, Rahul Nag, Claire Goldmann, Nicolas Ratel-Ramond, Cyrille Hamon, Marianne Impéror-Clerc

**Affiliations:** a Laboratoire de Physique des Solides, CNRS and Université Paris-Saclay, 91400 Orsay, France; b LPCNO, Université de Toulouse, INSA Toulouse, CNRS, UPS, 31077 Toulouse, France; Argonne National Laboratory, USA

**Keywords:** small-angle scattering, SAS, form factors, nanoparticles, data modeling

## Abstract

Since the morphology of nanoparticles directly influences many of their properties, accurately determining their shape is crucial for targeted applications. In this work, we focus on nanoprisms due to their widespread use and the limitations of direct imaging techniques in accurately describing their polygonal cross section. Specifically, we introduce a new tool for small-angle scattering (SAS) analysis of nanoprisms that requires minimal computation time compared with all-atom simulations and other form factor analyses. A key innovation in this work is the implementation of the Lebedev quadrature for isotropic averaging, which allows for accurate form factor calculations using few sampling points. This form factor model is developed for any *n*-sided prism and is compared with small-angle X-ray scattering and transmission electron microscopy experimental data for gold and/or silver nanoprisms (*n* = 3, 4, 5). For small sizes, the nanoprism form factor model is compared with the result obtained with the Debye equation from atomic coordinates, showing a very good agreement. We explore the effects of the aspect ratio and cross-sectional shape of the nanoprisms on the form factor curves and discuss the limitations of our approach. Overall, our method combines precise shape determination with rapid computation time, paving the way for detailed characterization of nanoprisms using SAS techniques, potentially even during their growth.

## Abstract 2

A new tool for form factor analysis of any *n*-sided nanoprism is presented. Shape analysis of various gold and/or silver nanoprisms (*n* = 3, 4, 5) is conducted using small-angle X-ray scattering experimental data. Our method compares well with transmission electron microscopy image analysis, providing additional details about the nanoprisms, and significantly reduces computation time compared with all-atom simulations.

## 1. Introduction

The functional properties of inorganic materials are governed by various factors, among which shape plays a pivotal role. For instance, the exposed crystalline facets of a nanocrystal determine reactivity in catalysis and can guide their self-assembly into functional materials, and, frequently, anisotropy gives rise to rich optical properties (Kinnear *et al.*, 2017 ▸; Baffou & Quidant, 2014 ▸; Jana, 2004 ▸; Reguera *et al.*, 2017 ▸). Accurately determining the shape of faceted nanoparticles is, therefore, a fundamental challenge in materials science. However, the conventional method, transmission electron microscopy (TEM), is not always ideal for characterizing complex 3D shapes, as its micrographs provide only 2D projections. Within the colloidal shape library, nanoprisms in the form of rods and platelets are found in a variety of materials, including metals, oxides, II–VI and III–V semiconductors, and perovskite nanocrystals (Dey *et al.*, 2021 ▸; Grzelczak *et al.*, 2008 ▸; Ghosh & Manna, 2018 ▸). For nanoprisms with triangular and square bases, it is usually feasible to infer the dimensions of the cross section from TEM image analysis. However, for nanoprisms with pentagonal, hexagonal and octagonal bases, this becomes significantly more complicated. In addition, nanoprisms show very often a preferred orientation on a TEM grid, preventing measurement of all their dimensions.

In this context, small-angle scattering (SAS) can provide detailed shape characterization, particularly when nanoparticles are uniformly dispersed. SAS offers a significant advantage over TEM by averaging data from a far larger number of particles, compared with the limited sample of around a hundred typically analyzed in TEM. For diluted colloidal dispersions, analyzing SAS data yields the form factor of the nanoparticles, averaged over all orientations. This form factor also appears in the scattering when nanoparticles are assembled together into supercrystals (Yager *et al.*, 2014 ▸). By modeling the form factor, it is possible to extract geometric parameters of the individual nanoparticles and predict the intensity of peaks in the structure factors of assemblies. However, nanoprisms are often approximated as spherocylinders, which imperfectly describe their polygonal cross section. Other models overlook the polygonal cross section of nanoparticles to save computational time (Lyu *et al.*, 2023 ▸). In general, SAS analysis of polyhedral shapes requires new models to better fit experimental data. Analytical formulas for volume integrals rapidly become complicated, even for simple shapes such as Platonic solids (Li *et al.*, 2011 ▸). The form factor of any particle shape can be obtained from the Debye formula (Debye, 1915 ▸) by calculating distances between all atomic pairs, but for large nanoparticles of a few tens of nanometres, computational time and resources are still an issue. Other approaches simplify volume integrals, either by reducing them to simpler volumes (Senesi & Lee, 2015 ▸; Croset, 2017 ▸; Yang *et al.*, 2023 ▸) or by using the divergence and Stokes theorems to first reduce the volume integral to an integral over the polyhedron’s faces and then further reduce it to integrals along the edges (Wuttke, 2021 ▸). The latter gives a general and elegant analytical expression of the form factor of any convex polyhedral shape, which optimizes computation time, but averaging over orientations is still needed.

The purpose of this work is to introduce a new tool for the SAS analysis of nanoprisms that requires minimal computation time. We utilize the expression derived by Wuttke (2021 ▸) for right prisms with regular polygonal cross sections and perform the orientation averaging using the Lebedev quadrature method. Specifically, the model is developed for any *n*-sided prism and is compared with small-angle X-ray scattering (SAXS) and TEM experimental data for gold and/or silver nanoprisms (*n* = 3, 4, 5). We examine the effects of the aspect ratio and cross-sectional shape of the nanoprisms on the form factor curves and discuss the limitations of our approach. The code is implemented in Python to facilitate reuse by other researchers. In the case of small nanoprisms, a very good agreement is found with a computation using the Debye equation from atomic coordinates. This work opens new avenues for the detailed characterization of nanoprisms using SAS techniques, potentially even during their synthesis.

## 2. SAS by *n*-sided prisms

**Figure 1:**
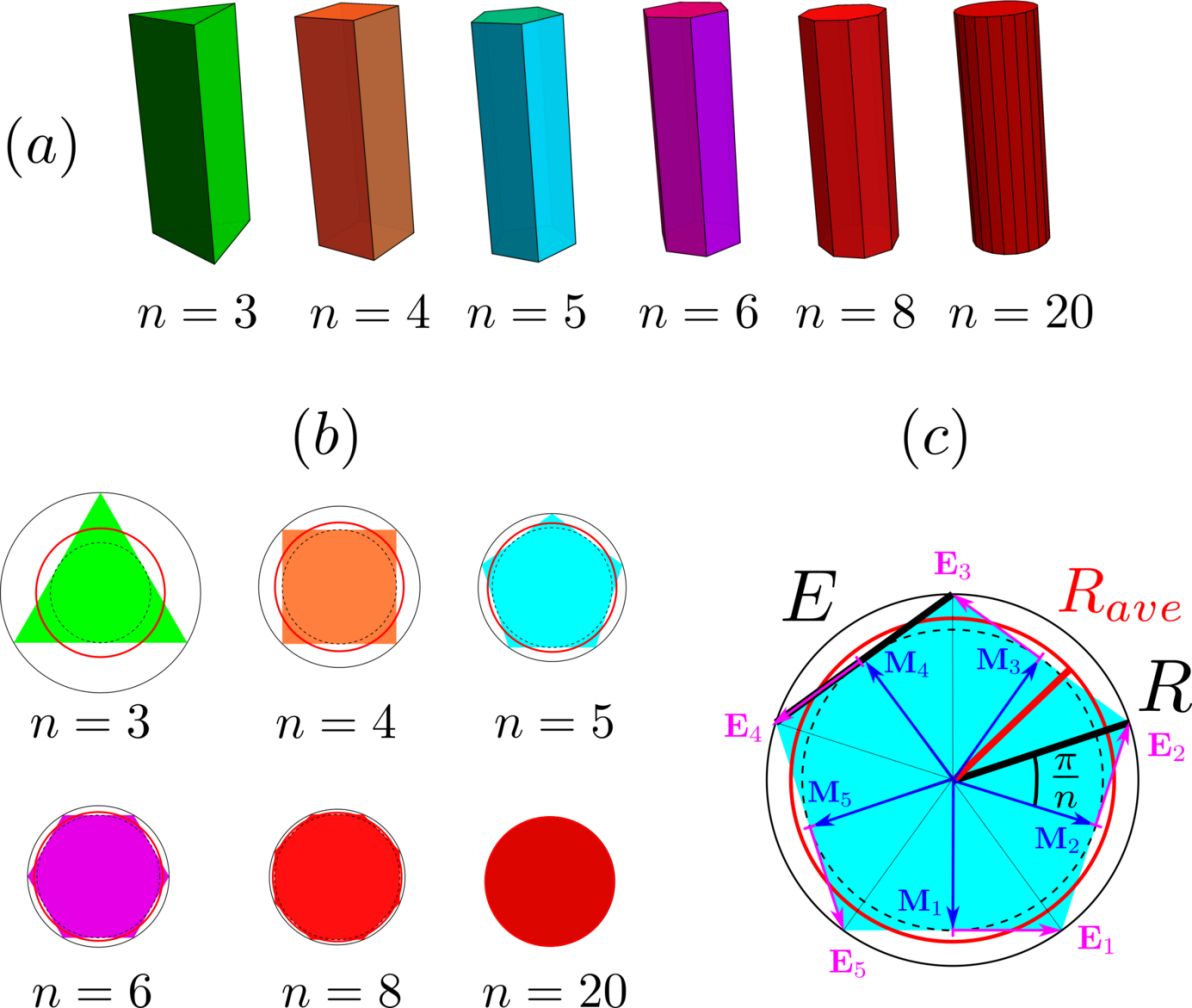
Right prisms with *n* sides. (*a*) Prisms with increasing *n* values at constant volume and fixed length *L* of aspect ratio γ = 3. Aspect ratio γ is defined as 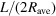. (*b*) The corresponding cross sections are regular polygons with identical areas. (*c*) Characteristic dimensions of the polygonal cross section illustrated for a pentagon (*n* = 5): *E* is the edge length, *R* is the circumradius of the regular polygon and 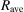 (in red) is the squared average radius. The arrows in dark blue and magenta correspond, respectively, to the vectors 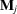 (middle of edges) and 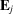 (half-edges) used in the form factor calculation in equation (5).

We consider particles having the shape of a right prism of length *L* and a cross section made of a regular polygon with *n* sides, as illustrated in Fig. 1 ▸(*a*). The size of a regular polygon can be characterized by its edge length *E* or by *R*, the circumradius of the polygon, with 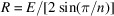. As shown in Fig. 1 ▸(*c*), the circumradius *R* is the radius of the circumcircle shown in black when the apothem 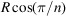 is the radius of the dashed circle. For comparison purposes, it is convenient to introduce an average radius 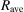,
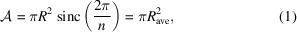
where 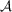 is the area of the polygon. 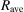 is the radius of the equivalent disc having the same area as the *n*-sided polygon and it is shown in red in Figs. 1 ▸(*b*) and 1 ▸(*c*). 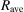 is also the squared average of the distance from the center of the polygon to any point of its perimeter,
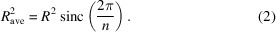
As expected, when the number of sides *n* increases, the cross section becomes closer and closer to a disc, and the difference between 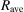 and *R* decreases, as illustrated in Fig. 1 ▸(*b*).

Anisotropic particles are often characterized by their aspect ratio γ. Here we define the aspect ratio of a right prism in a way that is independent of the number of sides *n* of the polygonal cross section: 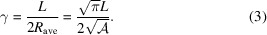
When 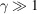, the prism shape describes a rod morphology, like in Fig. 1 ▸(*a*). In contrast, when 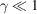, a platelet morphology is described by the same form factor. The influence of γ on the form factor curves is discussed in Section 4.4.

Note that, with this definition, the aspect ratio of a regular cube is 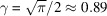 and not 1.

### 2.1. Form factor for a prism

Following Wuttke’s expression (Wuttke, 2021 ▸), the form factor *F*(**q**) for any right prism can be decomposed into the product of two factors, one for the component 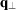 of the scattering vector **q** that is perpendicular to the cross section, and only depends on the length *L*, and another one, co-planar to the cross section, with the component 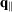, that depends on the number of sides *n* and the edge length *E* of the polygon. The perpendicular factor is
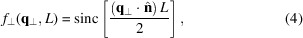
where 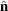 is the direction normal to the cross section. The length *L* gives rise to a standard sinc function for the form factor.

On the other hand, the parallel factor for a regular *n*-sided polygon of circumradius *R* can be expressed as
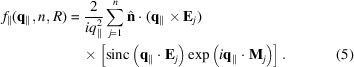
In the sum over all edges, 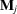 is the vector joining the center of the polygon to the middle of the *j*th edge and 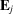 is the half-edge vector, as illustrated in Fig. 1 ▸(*c*) for a pentagon. The scattered intensity for one prism is
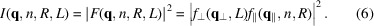
At scattering vectors close to zero, the limit of the scattered intensity 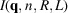 is 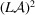, the squared volume of the particle.

### 2.2. Orientation average

In order to compare with experimental scattering curves of nanoparticle suspensions, we need to compute the isotropic form factor 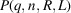 by calculating the orientation average of the intensity 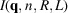 on all possible orientations of a particle. It is formally equivalent to the computation of the average of 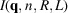 over the direction **u** of the scattering vector **q** at a fixed value of its modulus *q*: 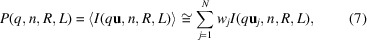
where 〈 〉 stands for the average of **u** over the unit sphere. Numerically, this average is computed as a discrete sum over a finite number *N* of sampling points of the unit sphere with their associated weights 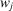, called a quadrature (Beentjes, 2015 ▸). Different choices are possible for the quadrature and numerical methods are still improving in this field (Beentjes, 2015 ▸; Gross & Atzberger, 2018 ▸). Depending on the choice of quadrature, the repartition of the sampling points on the unit sphere varies. We opted for the Lebedev quadrature (see Section 2.4) as a possible alternative to the more widespread Gauss–Legendre-based quadrature that couples a 1D Gauss–Legendre integration for the polar angle with constant steps for the azimuthal angle. The Lebedev quadrature is particularly interesting because it offers a much more uniform distribution compared with Gauss–Legendre. However, a limitation of the Lebedev quadrature is that the number of sampling points is fixed according to its order, with a maximum of only 5810 points for the highest solved order, 131. In contrast, the Gauss–Legendre quadrature allows for any number of sampling points, offering greater flexibility when more points are required, depending on the complexity of the scattering function. In this study, a quadrature order of 65 (1454 points on the unit sphere) is used by default to quickly calculate the isotropic average when adjusting parameters, and the resulting curve usually contains all the relevant features with a sufficient precision to compare with experimental data. The final result is computed with the maximum available order of 131 (5810 points on the unit sphere) for the quadrature.

### 2.3. Modeling scattering curves

A size distribution can be included for both the circum­radius *R* and the length *L* to model the scattering intensity. A Gaussian polydispersity is chosen, characterized by a standard deviation σ. To reduce calculation times, four options are implemented: polydispersity for *R* only, polydispersity for *L* only, the same polydispersity for both *R* and *L*, and distinct polydispersities for *R* and *L*. In practice, the final expression 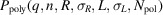 is computed as a simple sum for all options,
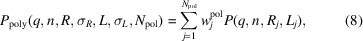
where *R* is the average value of the circumradius and 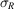 its standard deviation, *L* the average length and 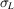 its standard deviation, and 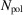 the number of values in the sum with weights 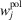, which are calculated differently depending on the option.

Experimental sets of data points 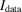 are adjusted by computing a model intensity 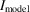 using either 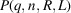 or 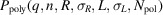 for the form factor. A reduced chi-squared 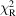 is computed for comparing different adjustments: 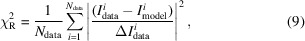
where 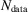 is the number of points in a data set, making the assumption that the error bars on experimental intensities are 10% of their absolute values with 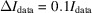.

### 2.4. Code implementation

A Python 3.10 code, tested under 3.12, has been written and is available at https://github.com/jules-marcone/prismformfactors and on *PyPI* (https://pypi.org/project/prismformfactors). An open-source Python package is used for the Lebedev quadrature (Lebedev, 1976 ▸; Filot, 2023 ▸). Polydispersity is implemented using the open-source *SASView* library (https://www.sasview.org/). Numerical results for a cylinder particle and for a prism with a square cross section are compared with those obtained using models available in *SASView*.

### 2.5. Debye equation approach from atomic coordinates

Scattering data can also be computed from atomic structural models constituted of atom types and coordinates using the well known Debye equation (Debye, 1915 ▸). In the case of particles made only of gold atoms, the Debye equation can be written as
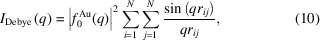
where *q* is the scattering vector magnitude, 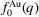 is the atomic form factor of a single gold atom, and 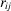 is the distance between atoms *i* and *j*.

In-silico structural models of pentagonal nanoprims have been created using the *ASE* Python library (Larsen *et al.*, 2017 ▸). The corresponding scattering data were further computed with the Debye equation using the *debyecalculator* Python library (Johansen *et al.*, 2024 ▸), which allows calculations on a GPU. In the case of structures larger than 60000 atoms, our nVidia Quadro GV100 GPU failed to compute the scattering intensity because of a lack of memory, illustrating the technical limitations in terms of computational power associated with the Debye approach. CPU calculations were performed with an 11th-generation Intel@Core i7-11850H@2.5GHzx16. Scattering data computed with the Debye equation (10) were then compared with those obtained with the form factor approach for nanoprisms of similar sizes and shapes.

To take into account the variation of the gold atomic form factor with the scattering vector at large *q* values, the same prefactor 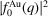 as in the Debye equation can be applied to the nanoprism form factor 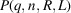 [equation (7)]. 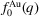 is computed using the tabulated Cromer–Mann coefficients. A constant gold atomic form factor 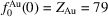 is a good approximation in the SAXS regime only until about 0.5 Å^−1^.

Finally, to facilitate comparison between the results using the Debye equation and the form factor approaches, scattered intensities can be scaled to 1 at 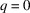 by dividing 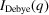 by a scaling factor of 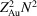, when 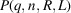 is divided by the square of the particle volume.

## 3. Materials and methods

### 3.1. Materials

Gold chloride trihydrate (HAuCl_4_·3H_2_O, ≥99.9%), silver nitrate (AgNO_3_, >99%), sodium borohydride (NaBH_4_, ≥96%), ascorbic acid (AA, ≥99%), hydrochloric acid (HCl, 37%), cetyltrimethylammonium chloride (CTAC, 25 wt% in H_2_O), cetyltrimethylammonium bromide (CTAB, ≥99%), benzyldimethylhexadecylammonium chloride (BDAC, 99%), sodium iodide (NaI, ≥99%) and trisodium citrate dihydrate (≥99%) were purchased from Merck. Milli-Q (MQ) water with a resistivity above 15 MΩ cm was used.

### 3.2. Synthesis of nanoparticles

Metallic nanoprisms (*n* = 3, 4 and 5) were synthesized as described previously (Goldmann *et al.*, 2023 ▸; Gómez-Graña *et al.*, 2013 ▸; Park *et al.*, 2011 ▸; Sánchez-Iglesias *et al.*, 2017 ▸; Marcone *et al.*, 2023 ▸).

#### 3.2.1. Gold triangular nanoprisms (C_3_NPs)

The synthesis of C_3_NPs first required the synthesis of gold seeds, followed by a seed-mediated growth. The seeds were prepared from a solution of CTAC (4.7 mL, 100 m*M*) and HAuCl_4_·3H_2_O (50 µL, 25 m*M*) in water, in which NaBH_4_ was added under vigorous stirring. After 30 s, the reaction mixture was kept undisturbed at room temperature for 120 min. For the C_3_NP growth, two separate solutions A and B were prepared simultaneously. The composition of solution A was MQ water (8 mL), CTAC (1.6 mL, 100 m*M*), HAuCl_4_·3H_2_O (80 µL, 25 m*M*), NaI (15 µL, 10 m*M*), AA (40 µL, 100 m*M*) and the composition of solution B was water (20 mL), CTAC (20 mL, 100 m*M*), HAuCl_4_·3H_2_O (1000 µL, 25 m*M*), NaI (300 µL, 10 m*M*), AA (400 µL, 10 m*M*). A portion of the aforementioned gold seed solution was diluted ten times in CTAC (100 m*M*), and 150 µL of this diluted solution was added quickly to solution A. Subsequently, 3.2 mL of solution A was rapidly mixed with solution B. The reaction mixture was gently stirred at room temperature for 2 h, centrifuged at 6000 rev min^−1^ for 15 min and washed before purification by depletion in CTAC (110 m*M*) overnight. After the shape separation, the precipitate was collected and washed with 2.5 m*M* CTAC and stored in 2.5 m*M* CTAC.

#### 3.2.2. Gold/silver square nanoprisms (C_4_NPs)

This synthesis required three steps: the fabrication of monocrystalline gold seeds, followed by a first seed-mediated growth to obtain gold nanorods, and finally a second seed-mediated growth of silver to obtain the core–shell gold/silver C_4_NPs. The gold seeds were obtained from a solution of CTAB (50 m*M*) and decanol (14 m*M*) in H_2_O (7 mL), to which was added HAuCl_4_ (140 µL, 25 m*M*), AA (35 µL, 100 m*M*) and NaBH_4_ (280 µL, 20 m*M*). The mixture was stirred at room temperature for 1 h. The gold nanorods were obtained from 100 mL of a similar CTAB/decanol/water solution, to which was added AgNO_3_ (800 µL, 10 m*M*), HCl (7 mL, 1 *M*), HAuCl_4_ (2 mL, 25 m*M*), AA (1.3 mL, 100 m*M*) and 6 mL of the previously prepared seeds solution. The mixture was stirred at 28°C for 4 h, before being centrifuged at 13000 rev min^−1^ for 1 h and washed with water twice. The C_4_NPs were obtained from a solution of 250 mL of a similar CTAB/decanol/water solution, to which was added AgNO_3_ (500 µL, 100 m*M*), HAuCl_4_ (5 mL, 25 m*M*), AA (2 mL, 100 m*M*), HCl (25 mL, 1 *M*) and the previously prepared nanorod solution (650 µL, [Au] = 8.75 m*M*). The solution was stirred at 28°C for 4 h, after which the C_4_NPs were collected and washed by centrifugation.

#### 3.2.3. Gold/silver pentagonal nanoprisms (C_5_NPs)

This synthesis required three steps: the fabrication of pentatwinned gold seeds, followed by a first seed-mediated growth to obtain large gold decahedra, and a second seed-mediated growth of silver to obtain the core–shell gold/silver C_5_NPs. The seeds were synthesized in a solution of CTAC (60 m*M*) in water (33 mL) to which was added trisodium citrate (4 mL, 50 m*M*) at 30°C. The mixture was left at 30°C for 30 min before adding NaBH_4_ (1 mL, 25 m*M*). After stirring for 30 s, the mixture was left for 5 days in an oven at 40°C to obtain pentatwinned seeds. The gold decahedra were prepared from a solution of BDAC (100 m*M*) in water (400 mL) to which was added HAuCl_4_ (8 mL, 25 m*M*) and AA (3 mL, 100 m*M*). A portion of the seed solution (1200 µL, [Au] = 0.3 m*M*) was added under fast stirring. The mixture was subsequently left under slow stirring at 30°C for 30 min. The decahedra were then centrifuged for 10 min at 6000 rev min^−1^ and washed with water, before being stocked in 5 mL of 5 m*M* CTAC. The C_5_NPs were synthesized from a solution of decahedra ([Au] = 0.25 m*M*) and CTAC (10 m*M*) in water (100 mL) to which was added, at 70°C under fast stirring, AgNO_3_ (1.25 mL, 100 m*M*) and AA (5 mL, 100 m*M*). The mixture was left under stirring at 70°C for 2 h before purification by successive depletions at 60 and 40 m*M* of CTAC. The C_5_NPs were then stored in 5 m*M* CTAC.

### 3.3. TEM

TEM was performed at Imagerie-Gif (I2BC CNRS, Gif-sur-Yvette, France) using a JEOL JEM-1400 microscope operating at 120 kV with a filament current of ≈55 µA.

### 3.4. SAXS

SAXS measurements were performed on the SWING beamline of the SOLEIL synchrotron (Saint-Aubin, France) at a beam energy of *E* = 16 keV with a sample-to-detector distance of 6.22 m, resulting in a *q* range of 0.001 to 0.24 Å^−1^. The beam size was approximately 500 × 200 µm (horizontal × vertical). All measurements were performed at room temperature (295 K). The scattered signal was recorded by an Eiger 4M detector (Dectris Ltd, Switzerland) with a pixel size of 150 µm (2 × 2 binning mode). Preliminary data treatment (angular averaging and normalization) was done using the software *Foxtrot* developed at the beamline (https://www.synchrotron-soleil.fr/fr/lignes-de-lumiere/swing), which yielded the intensity as a function of the scattering vector magnitude *I*(*q*) in absolute units. Complementary ultra-small-angle X-ray scattering (U-SAXS) was conducted on the ID02 SAXS beamline of ESRF (Grenoble, France), with a beam energy of *E* = 12 keV and a sample-to-detector distance of 36 m, thus allowing ultra-small *q* values down to ≈2 × 10^−4^ Å^−1^ to be reached. Form factor scattering curves were measured on diluted aqueous suspensions of the different metallic nanoparticles and the signal from the appropriate diluted CTAC solution was subtracted from the raw signal.

## 4. Results and discussion

### 4.1. Influence of the number of sides *n*

**Figure 2:**
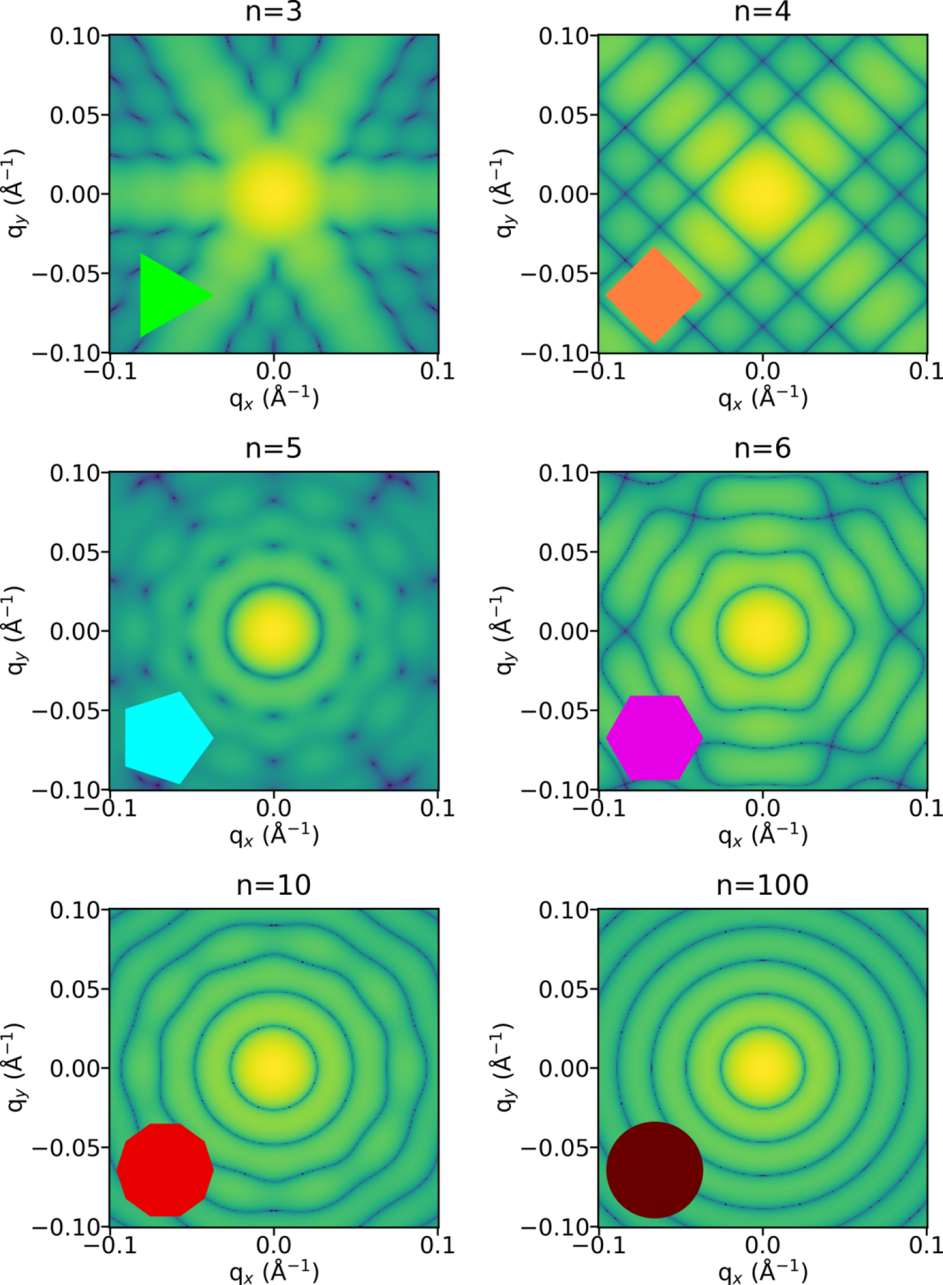
Comparison of the scattered intensity in the cross-section plane for different *n*-prisms (*n* = 3, 4, 5, 6, 10 and 100) with the same circumradius *R* = 15 nm. The cross section is shown at the bottom left of each 2D plot.

In Fig. 2 ▸, the scattered intensity in the cross-section plane 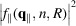 [see equations (5) and (6)] is plotted to compare different particles with the same circumradius *R*. As expected, the effect of the polygonal shape is very strong for small values of *n*, along with a strong even–odd effect. For all odd values of *n*, the symmetry order of the 2D plot is 2*n*. It is visible in Fig. 2 ▸ for the triangular cross section with a sixfold symmetry and for the pentagonal cross section with a tenfold symmetry. This comes from the fact that the scattered intensity always has a symmetry center at the origin of the reciprocal space, a general relation known as the Friedel law. For values of *n* larger than 10, the anisotropy in the cross-section plane becomes much less pronounced and it is hard to discern the difference from the cross section of a cylinder.

**Figure 3:**
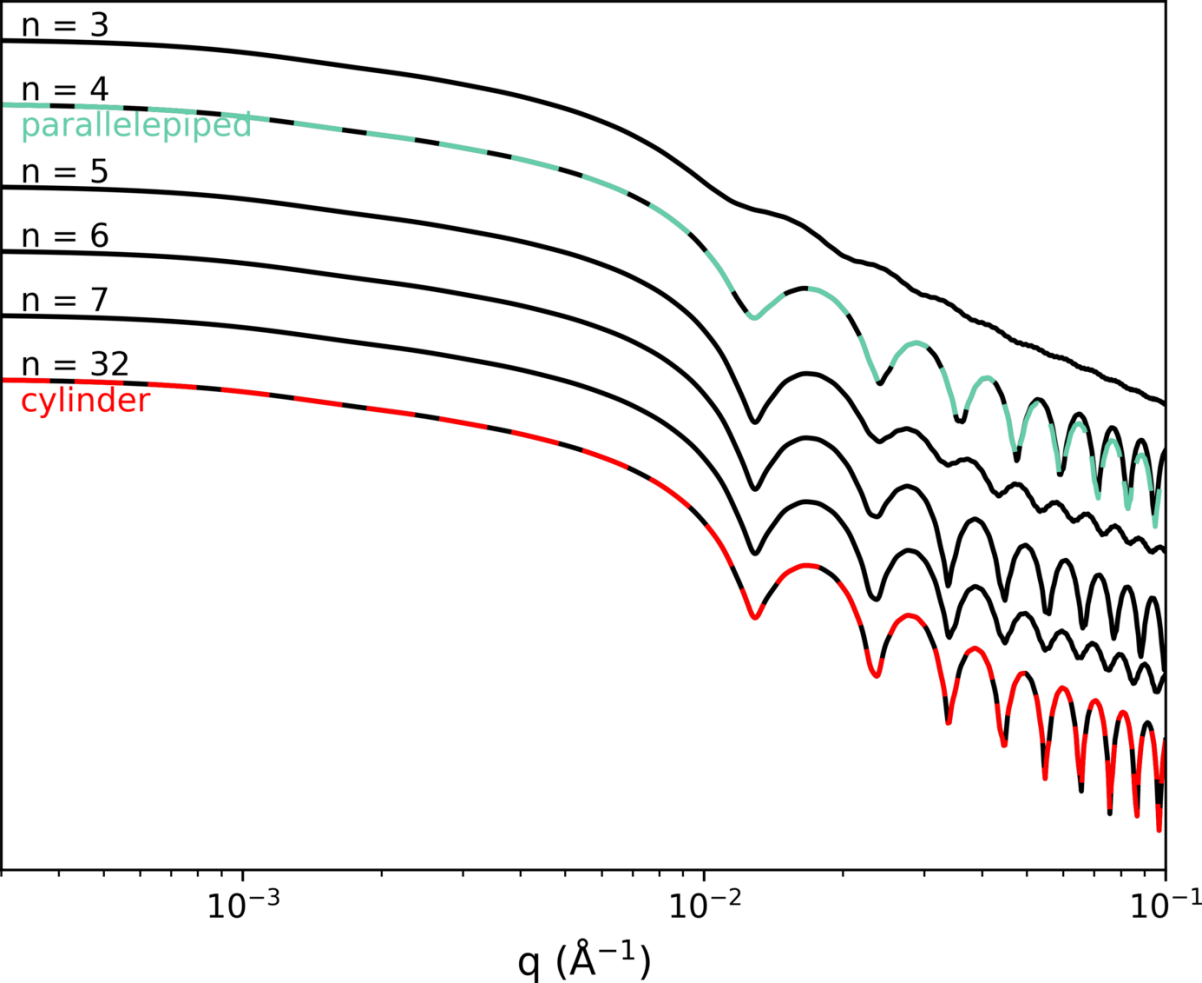
Isotropic form factor 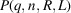 of various *n*-sided prisms for *n* ranging from 3 to 32 computed with the Wuttke formula adapted for prisms [see equation (6)]. All particles have the same length *L* = 450 nm and cross-section area (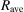 = 30 nm) with an aspect ratio γ = 7.5. The curves are compared with the calculation using the standard expressions of a parallelepiped (in light blue) and a cylinder (in red). Curves are shifted along the vertical axis for a better comparison.

The same even–odd effect is visualized in Fig. 3 ▸ on the isotropic form factors 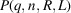 [see equation (7)] when varying *n* at constant volume and length as depicted in Fig. 1 ▸. The zeros in the oscillation regime at higher *q* values are much less pronounced for an odd number of sides than for an even one. The relative positions of the maxima of these oscillations are different depending on *n*, giving a signature of the polygonal cross section. In addition, as a verification, the form factor for *n* = 4 is superimposed on the usual parallelepiped model (light blue curve), while for *n* = 32, the usual cylinder model (red curve) gives the same result. We conclude that the *n*-prism particles have a clear signature in their isotropic form factor for values of *n* less than 8. Triangular and pentagonal prisms are easily recognized from the damping of the oscillations at large *q* values. For *n* values larger than 8, it is not possible in practice to discriminate them from a cylinder model, as all the oscillations are superimposed in a realistic *q* range for measurements.

### 4.2. Combining with TEM in the case of cuboids

We perform a first test of the nanoprism form factor model by considering metallic nanoprisms with a square cross section (C_4_NPs) (see Section 3.2). This simple shape is a good starting point to compare with experimental data as the model is equivalent to the well established expression of the form factor for a parallelepiped model, as discussed in Section 4.1 and shown in Fig. 3 ▸.

**Figure 4:**
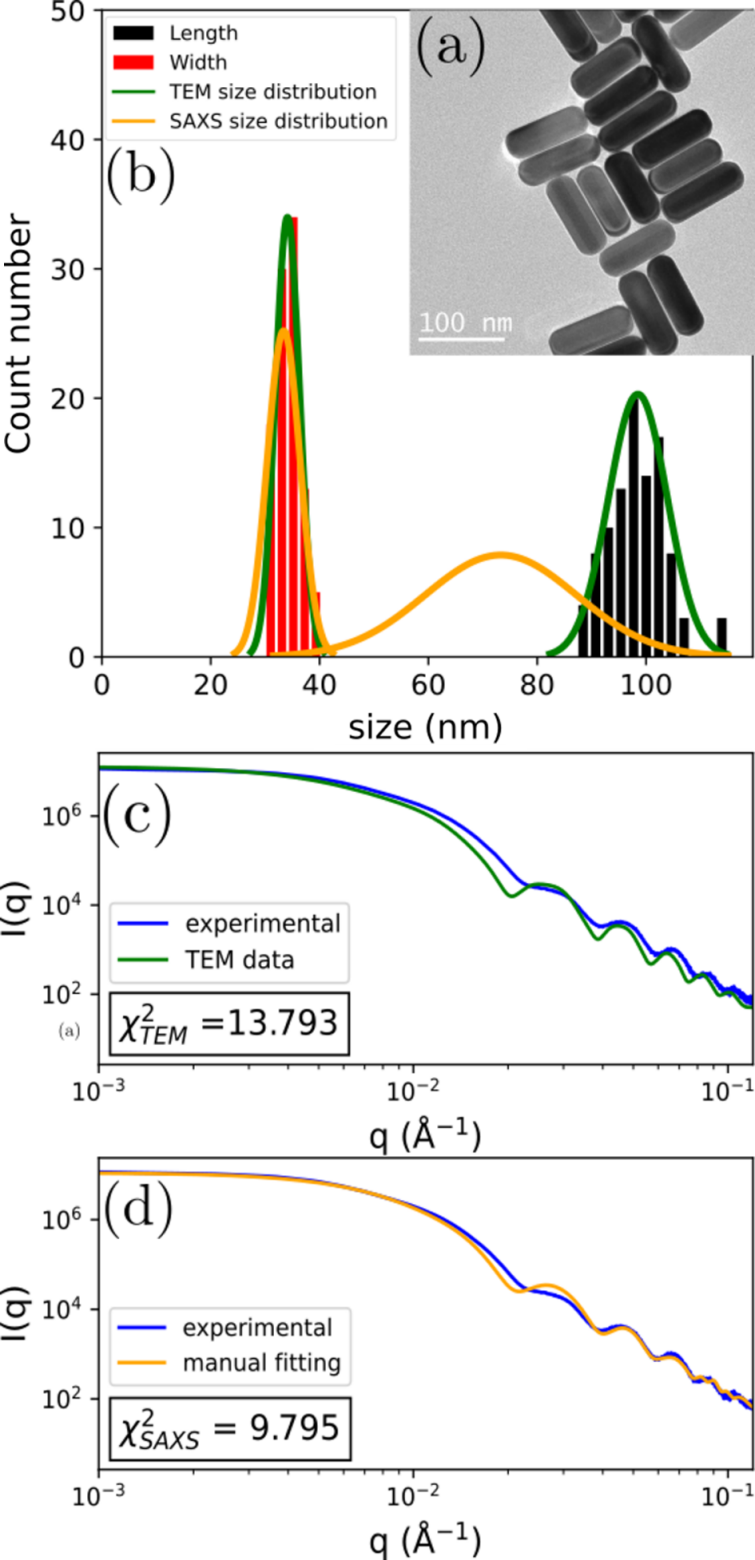
Study of a population of four-sided prisms in both TEM and SAXS. (*a*) TEM image of the C_4_NPs. (*b*) Histograms of the particle width (red) and length (black) determined by 100 measurements in TEM. Gaussian distributions of the size parameters determined by TEM only (green) and by manual fitting of the experimental model (orange). (*c*) Comparison of the experimental SAXS data (blue) and the experimental model with the parameters found in the TEM analysis (green). (*d*) Comparison of the experimental SAXS data (blue) and the experimental model with manually fitted parameters (orange).

The two dimensions (width and length) of C_4_NPs can be easily measured using TEM imaging as the particles generally lie flat when deposited on a TEM grid, as shown in Fig. 4 ▸(*a*). The histogram of width (equivalent to the edge for four-sided prisms) and length measured for 100 particles [see Fig. 4 ▸(*b*)] gives *L*_TEM_ = 98 nm and *E*_TEM_ = 34 nm with a polydispersity of 0.05 for both dimensions. We can compare these with the experimental SAXS scattering curve [see Fig. 4 ▸(*c*)] measured on a dilute suspension of the same batch of C_4_NPs by calculating the form factor corresponding to these TEM values. Here, initially, a global agreement was found but with some remaining discrepancies. A manual adjustment (see Section 2.3) of the dimensions with the model is then done to match the experimental curve as best as possible [see Fig. 4 ▸(*d*)]. Experimentally, the size distribution for the edge length is usually narrow, while the size distribution for length is broader. By adjusting the simulated curve to the experimental oscillations in the form factor, the value of the edge length is thus refined to *E* = 33 nm with a polydispersity of 0.09. Meanwhile, adjusting the simulation to the experimental curve at low *q* values yields an average length value of *L* = 73 nm with a large polydispersity of 0.19.

This analysis with our model shows that TEM imaging on a small number of particles underestimates the polydispersity on both dimensions. When the edge length values from both methods are in good agreement, the broadness of the length distribution (0.19 polydispersity) is reliably obtained only from the SAXS analysis. The final adjustment [see Fig. 4 ▸(*d*)] still shows discrepancies with the experimental curve, indicating that a small amount of another nanoparticle shape might be present in the suspension as a by-product of the synthesis.

### 4.3. Pentagonal nanoprisms – determination of the number of sides

Since pentagonal nanoprisms (C_5_NPs) are typically synthesized from pentatwinned seeds (see Section 3.2), it is known from previous studies (Johnson *et al.*, 2008 ▸) that their cross section is pentagonal. However, the relation between the width measured for a pentagonal nanoprism deposited on a TEM grid and its radius or edge length depends on the orientation of the cross section relative to the grid, making it challenging to precisely evaluate its size. In this context, the form factor analysis provides a much more accurate determination of nanoprism dimensions.

**Figure 5:**
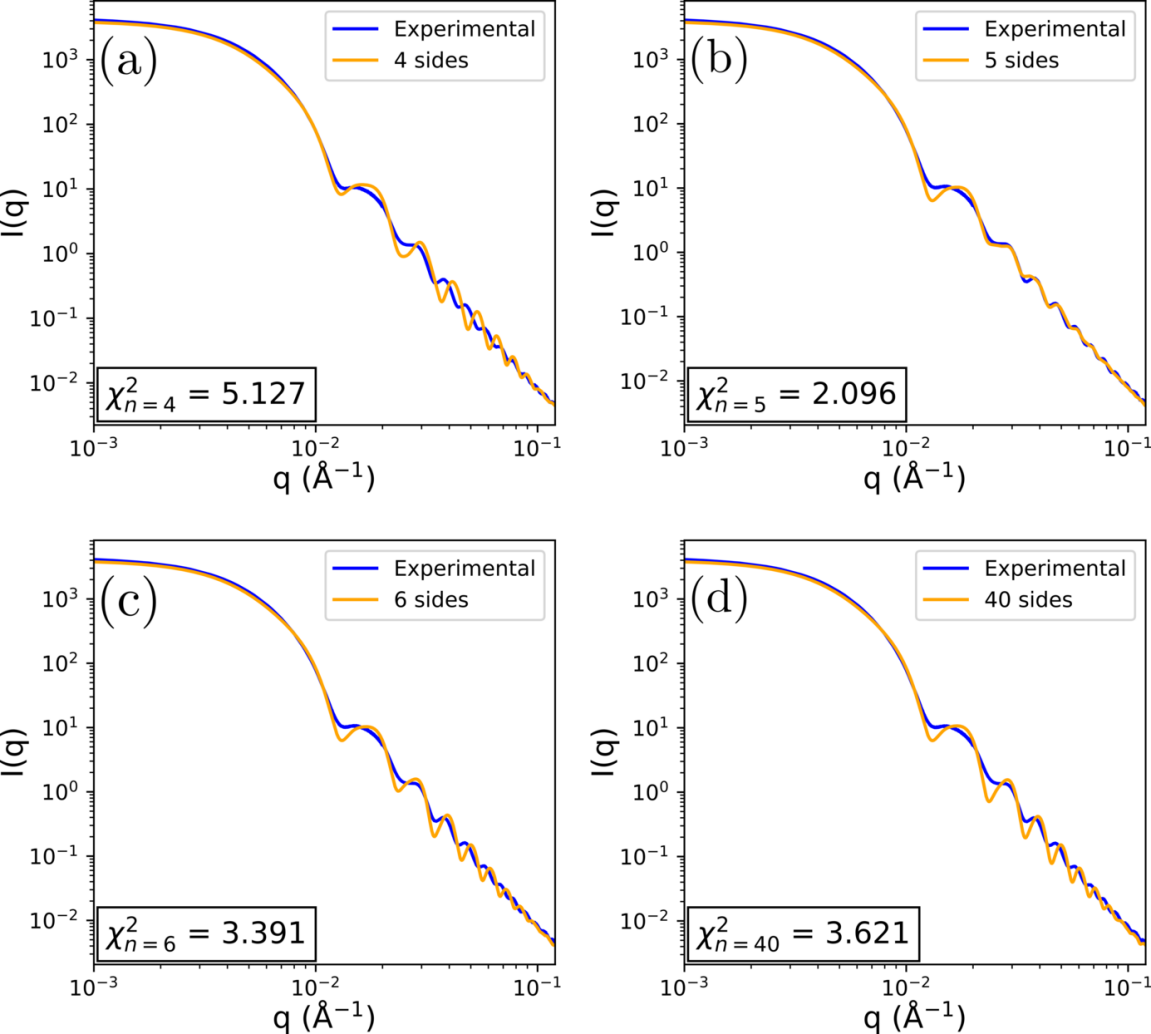
Comparison of an experimental form factor of C_5_NPs with form factor models of varying number of sides. (*a*) Four sides. (*b*) Five sides. (*c*) Six sides. (*d*) 40 sides, approximating a cylinder.

In Fig. 5 ▸, a typical experimental scattering curve for a batch of C_5_NPs is analyzed. This curve has a remarkable number of well defined oscillations at large *q* values, a good indication that the particles have a low size polydispersity. The analysis using the nanoprism form factor model is performed for *n* = 4, 5, 6 and 40, the last value corresponding to an effective circular cross section. Only *n* = 5 [Fig. 5 ▸(*b*)] is able to reproduce the exact positions of all oscillations. For other values of *n*, the positions exhibit a shift: if an adjustment is made based on the position of one particular oscillation, the positions of the others do not match the experimental curve. Collectively, this analysis shows that this batch contains a large majority of pentagonal nanoprisms.

The form factor model for *n* = 5 gives accurate dimension values of the pentagonal nanoprisms with 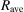 = 30 ± 1.5 nm and *L* = 117 ± 6 nm, giving an aspect ratio γ of 2.0 for this particular nanoprism batch. These dimensions are compatible with TEM imaging performed on 100 particles (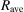 = 30 nm and *L* = 135 nm), but most importantly this analysis demonstrates that the size distributions are very narrow, with a polydispersity value of only 0.05 for both dimensions. Finally, as with the previous particles with a square cross section (Section 4.2), the discrepancy around the first oscillation with the experimental curve might be attributed to a tiny amount of synthesis by-products. Another explanation might be that the cross section is not a perfect regular pentagon, or the presence of caps at the extremities of the particles.

Note here that, with an aspect ratio value γ = 2.0, the oscillations in the form factor are governed not only by the average radius 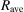 but also by the length *L*. This brings us to an important feature, the influence of the aspect ratio on the form factor.

### 4.4. Influence of aspect ratio

**Figure 6:**
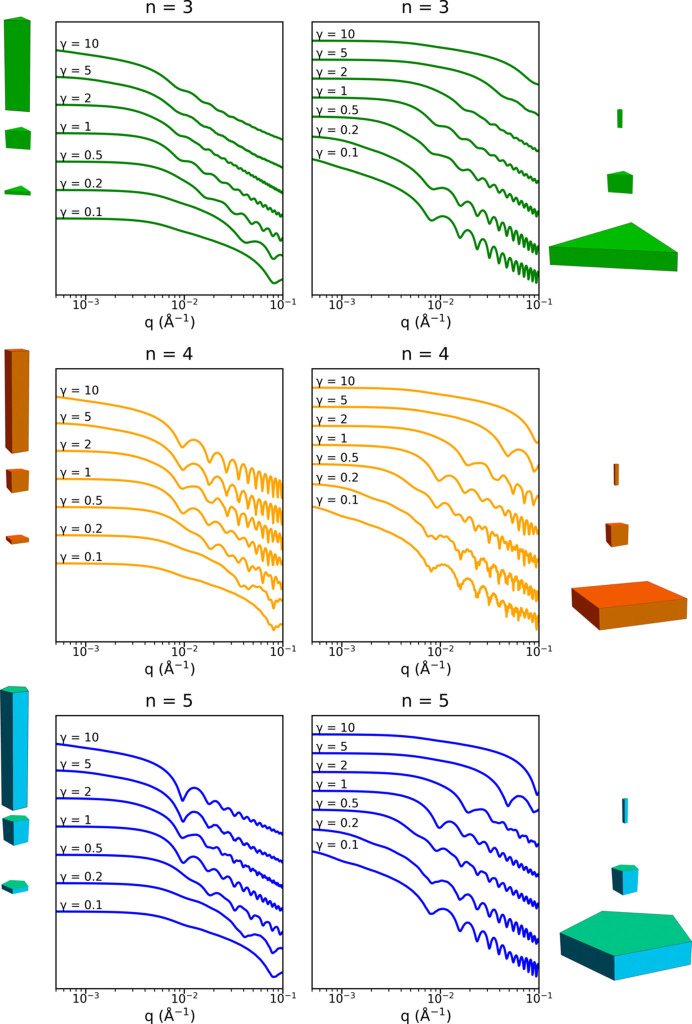
Isotropic form factor 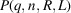 for three-, four- and five-sided prisms with the same cross-section area on the left (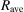 = 40 nm) and the same length on the right (*L* = 80 nm) and different aspect ratios γ.

In Fig. 6 ▸, the scattered intensity for three-, four- and five-sided prisms is plotted for different aspect ratios ranging from a thin platelet morphology for γ = 0.1 to a long rod morphology for γ = 10. On the left-hand side of the figure, the cross-section area is fixed (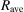 = 40 nm) and only the length *L* changes, while on the right-hand side, the length *L* is fixed at 80 nm when varying the cross-section area. These two situations may correspond, for example, to the evolution of the scattering curves during the growth of nanoprisms in solution, helping to discriminate which dimension is increasing the most. For aspect ratios around 1, the scattered intensity exhibits a direct transition from a plateau regime at low *q* values to a regime where many oscillations are present, related to the fact that the two characteristic dimensions 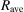 and *L* have similar values. When the objects have a large anisotropy, like platelets (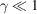) or rods (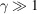), the oscillations are dominated by the smallest dimension, either the thickness for a platelet or the section for a rod, while the larger dimension controls the behavior of the scattering curve at low *q* values. The even–odd effect is again observed, with more pronounced oscillations for *n* = 4 than for *n* = 3 and *n* = 5. The typical nanoprism dimension in Fig. 6 ▸ is 40 nm, which corresponds to a characteristic value for metallic nanoprisms; this gives rise to a large number of oscillations in the small-angle *q* range, which makes it possible to distinguish the number of sides *n*. However, when the overall size gets smaller, like for rods with γ = 10, the oscillations are shifted to *q* values larger than 0.1 Å^−1^. In that case, in order to discriminate the value of *n* from the positions of the oscillations, measurements have to be performed at larger *q* values where the atomic form factor is no longer constant. This means that the atomic structure inside a nanocrystal should be considered when analyzing particles of a few nanometres in size, as further discussed in Section 4.6.

### 4.5. Triangular platelets – finding a non-visible dimension in TEM

**Figure 7:**
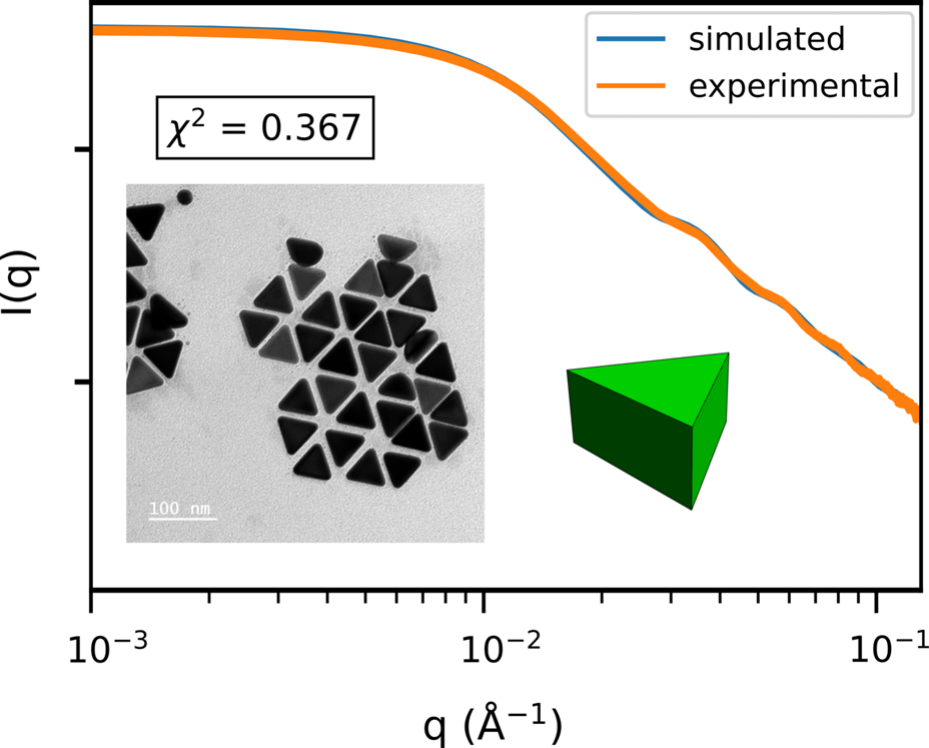
Triangular thick platelets (C_3_NP). Average edge length is 59.6 nm, average thickness is 27.9 nm, with a linked polydispersity for both dimensions of 0.13. The 3D shape (in green color) illustrates these dimensions, with a corresponding aspect ratio of γ = 0.63.

The case of triangular platelets corresponds to *n* = 3 with an aspect ratio 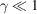. In Fig. 7 ▸, an experimental form factor scattering curve is shown for C_3_NPs (see Section 3.2 for synthesis aspects) and is compared with a triangular platelet form factor model, which shows a very good agreement, with a 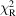 value of around 0.4. A representative TEM image for the same C_3_NP sample is displayed in Fig. 7 ▸, and image analysis performed over 118 different particles yields an average size 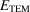
 = 57.7 ± 3.3 nm for the edge length. An important observation is that platelet particles consistently lie flat on the TEM grid, making it impossible to measure their thickness directly by imaging. In contrast, SAXS curve analysis provides both the edge length and thickness with high accuracy. From the SAXS curve modeling, we can deduce the size distribution both for the edge length with *E* = 59.6 ± 7.7 nm and for the thickness with *L* = 27.9 ± 3.6 nm. Here the polydispersity value is 0.13 for both dimensions. The average aspect ratio is γ = 0.63, showing that such platelets are rather thick, in agreement with previous studies (Goldmann *et al.*, 2023 ▸; Scarabelli *et al.*, 2014 ▸; Kuttner *et al.*, 2018 ▸). Although the effective shape of such nanoparticles is a beveled nanoprism (Kim *et al.*, 2017 ▸), it can be described well by our regular nanoprism model. In conclusion, SAXS analysis provides dimensional information, such as thickness, which is challenging to obtain through standard TEM image analysis.

### 4.6. Smaller objects and size limit

When the dimensions of the nanoprisms are small, typically less than about 10 nm for both dimensions, it is possible to test the limitations of the form factor model for small sizes. To do so, small pentagonal nanoprisms (a few thousand atoms) can be modeled using two different methods, the form factor approach and the Debye equation (see Section 2.5). We have considered pentagonal nanoprisms with an edge length of 1.55 nm, corresponding to four times the interatomic Au–Au distance, and with a variable aspect ratio (γ = 2.2, 2.7, 3.5 and 5.6). Detailed dimensions of the resulting particles are shown in Table 1 ▸.

**Figure 8:**
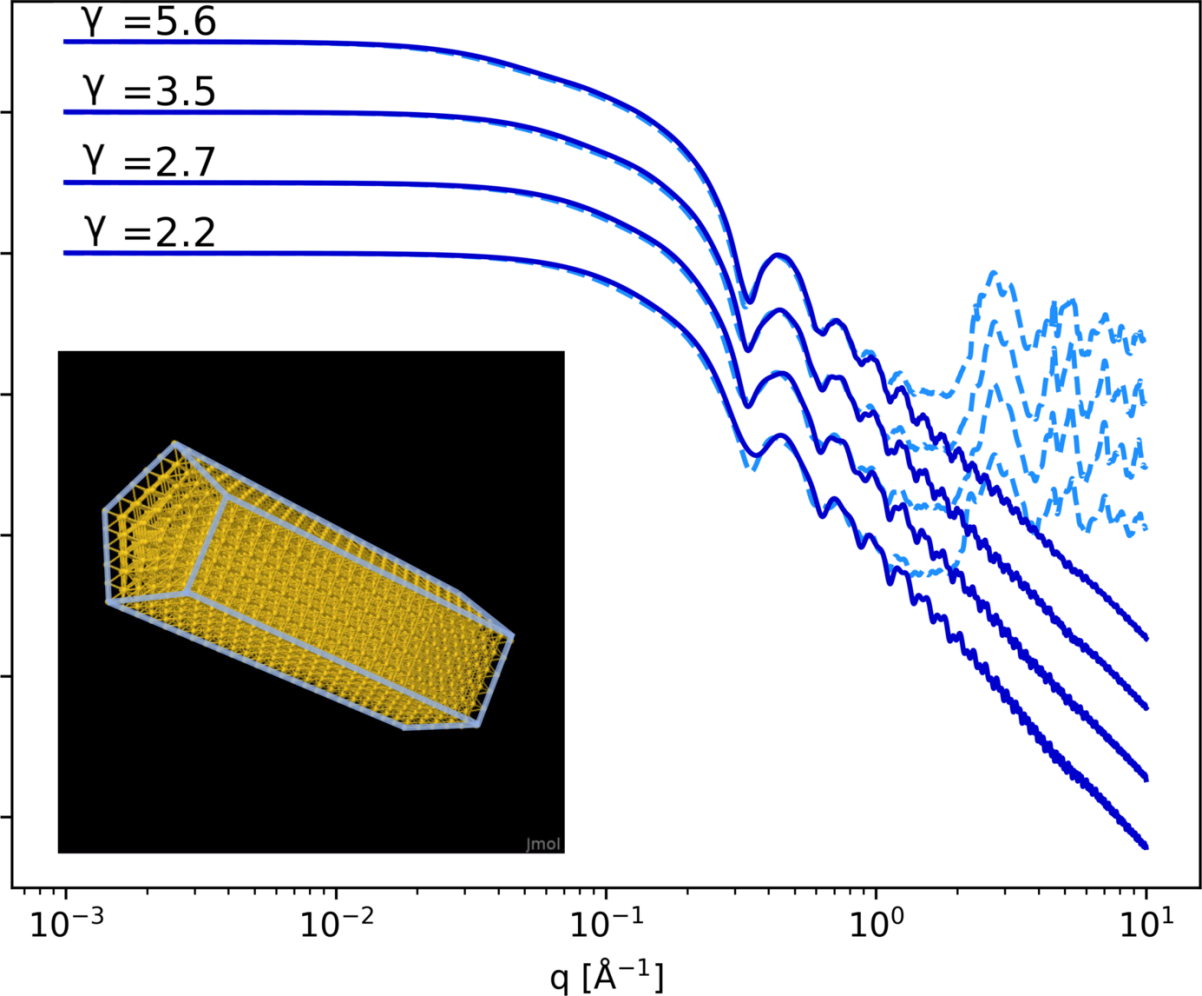
Calculated form factor (solid lines) compared with the Debye equation (dashed lines) for small C_5_NPs with an edge length of 1.55 nm and four different aspect ratios γ = 2.2, 2.7, 3.5 and 5.6 (see Table 1 ▸). Form factor and Debye expressions are both scaled to one at *q* = 0 for each scattering curve.

Fig. 8 ▸ shows the results obtained with the form factor and the Debye computation approaches. In the SAXS region (*i.e.q* < 0.5 Å^−1^), both methods show a very good agreement. However, as *q* increases, the atomic scale structure of gold is revealed in the Debye calculation, while it is not accounted for in the form factor approach.

From a computational point of view, the use of the Debye equation is much more demanding than the form factor approach, passing from 450 ms in the form factor case to a few seconds (33.6 s for 3876 atoms) per iteration in the Debye case (CPU time). In addition, an increase in the number of atoms increases the computation time in the case of the Debye calculation, while the computation time remains constant with the form factor approach.

In the case of particles of even smaller sizes, typically around 1 nm of edge size, one should expect the observation of one or two oscillations in the form factor, in a *q* region approaching the domain where atomic scale structure becomes dominant (*q* > 0.5 Å^−1^). Since our method does not account for the signal arising from the atomic structure, using the Debye equation is preferable for simulating the scattering signal in such cases. However, for larger particles (above 10 nm), the form factor approach is recommended, as it provides equivalent results with significantly faster computation times.

## 5. Conclusion

In this work, we introduce a new tool for the SAS analysis of *n*-sided nanoprisms that requires minimal computation time. We utilize the expression derived by Wuttke (2021 ▸) for right prisms with regular polygonal cross sections and perform the orientation average using the Lebedev quadrature method, which allows for accurate form factor calculations using few sampling points. A very good agreement is obtained when comparing our model’s results with the Debye equation approach from atomic coordinates for test particles containing only a few thousand atoms.

Comparison with TEM experimental data for gold and/or silver nanoprisms (*n* = 3, 4, 5) illustrates how the shape and dimensions are measured much more accurately and completely using SAXS analysis rather than TEM imaging, like in determining the thickness of triangular platelets, the number of sides of a population of nanoprisms or the exact average radius of pentagonal rods.

In the future, less regular cross sections could be easily implemented in the model, to take into account truncation effects along edges of a particle. This work also opens new avenues for the detailed characterization of nanoprisms using SAS techniques, potentially even during their synthesis.

## Figures and Tables

**Table 1:** Dimensions of the atomic structural models of C_5_NPs used for the comparison between Debye and form factor approaches The edge length is fixed at *E* = 1.55 nm.

Aspect ratio γ	2.2	2.7	3.5	5.6
Length (nm)	4.95	6.05	7.98	12.65
No. of gold atoms	1540	1869	2477	3876
